# Postnatal Effects of Sex Hormones on Click-Evoked Otoacoustic Emissions: A Study of Adolescents with Gender Dysphoria

**DOI:** 10.1007/s10508-020-01652-8

**Published:** 2020-02-13

**Authors:** Sarah M. Burke, Jason O. van Heesewijk, Willeke M. Menks, Daniel T. Klink, Baudewijntje P. C. Kreukels, Peggy T. Cohen-Kettenis, Julie Bakker

**Affiliations:** 1 Department of Medical Psychology, Center of Expertise on Gender Dysphoria, Amsterdam University Medical Centers, 1081 HX Amsterdam, The Netherlands; 2 Department of Developmental and Educational Psychology, Brain and Development Research Center, Leiden University, Leiden, The Netherlands; 3 Donders Institute for Brain, Cognition and Behaviour, Radboud University, Nijmegen, The Netherlands; 4 Department of Pediatric Endocrinology, Center of Expertise on Gender Dysphoria, Amsterdam University Medical Centers, Location VUmc, Amsterdam, The Netherlands; 5 Pediatrics and Genetics Research Unit, Division of Pediatric Endocrinology, Department of Pediatrics, Ghent University Hospital, Ghent University, Ghent, Belgium; 6 GIGA Neuroscience, University of Liege, Liège, Belgium

**Keywords:** Click-evoked otoacoustic emissions, Estradiol, Testosterone, Gender dysphoria, Gonadotropin-releasing hormone analogs, Sex differences

## Abstract

Click-evoked otoacoustic emissions (CEOAEs) are echo-like sounds, generated by the inner ear in response to click-stimuli. A sex difference in emission strength is observed in neonates and adults, with weaker CEOAE amplitudes in males. These differences are assumed to originate from testosterone influences during prenatal male sexual differentiation and to remain stable throughout life. However, recent studies suggested activational, postnatal effects of sex hormones on CEOAEs. Adolescents diagnosed with gender dysphoria (GD) may receive gonadotropin-releasing hormone analogs (GnRHa) in order to suppress endogenous sex hormones and, therefore, pubertal maturation, followed by cross-sex hormone (CSH) treatment. Using a cross-sectional design, we examined whether hormonal interventions in adolescents diagnosed with GD (62 trans boys, assigned female at birth, self-identifying as male; 43 trans girls, assigned male at birth, self-identifying as female), affected their CEOAEs compared to age- and sex-matched controls (44 boys, 37 girls). Sex-typical differences in CEOAE amplitude were observed among cisgender controls and treatment-naïve trans boys but not in other groups with GD. Treatment-naïve trans girls tended to have more female-typical CEOAEs, suggesting hypomasculinized early sexual differentiation, in support of a prominent hypothesis on the etiology of GD. In line with the predicted suppressive effects of androgens, trans boys receiving CSH treatment, i.e., testosterone plus GnRHa, showed significantly weaker right-ear CEOAEs compared with control girls. A similar trend was seen in trans boys treated with GnRHa only. Unexpectedly, trans girls showed CEOAE masculinization with addition of estradiol. Our findings show that CEOAEs may not be used as an unequivocal measure of prenatal androgen exposure as they can be modulated postnatally by sex hormones, in the form of hormonal treatment.

## Introduction

Otoacoustic emissions (OAEs) are sound waves that are produced in the cochlea and propagate back through the middle ear into the external ear canal. OAEs that appear in the absence of any external stimulus are called spontaneous OAEs (SOAEs). When OAEs occur in response to brief transient click-stimuli, they are called click-evoked OAEs (CEOAEs) (Kemp, 2002; Rodenburg & Hanssens, 1998). Generally, females have stronger CEOAEs and more numerous SOAEs compared with males (McFadden, Loehlin, & Pasanen, 1996), and this sex difference in emission strength and frequency has repeatedly been observed in neonates (Burns, Hoberg Arehart, & Campbell, 1992; Morlet et al., 1995; Strickland, Burns, & Tubis, 1985). Therefore, it is assumed that the sex difference in OAE amplitude develops during the prenatal sexual differentiation of the fetus. It is thought that this fetal sexual differentiation is a result of organizational effects of sex hormones on a developing brain, causing it to become more female- or male-typical (Bao & Swaab, 2011). According to the organizational-activational hypothesis, these previously organized brain structures are activated by differences in sex hormone levels later in life, especially from puberty onwards. Although OAEs are reported to be relatively constant throughout life (Burns, 2009, 2017; McFadden et al., 1996), they might also be activated later in life. Several animal studies support the notion that the weaker CEOAEs present in males originate from relatively high prenatal exposure to androgens (for review, see McFadden, 2008; McFadden, Pasanen, Valero, Roberts, & Lee, 2009). In addition, OAE studies in twins showing that women who shared the uterus with a male co-twin had “masculinized” OAEs (McFadden, 1993; McFadden et al., 1996), provide indirect evidence for the dampening, organizational effects of androgens on the human auditory system.

Individual differences in OAEs have been shown to be relatively stable throughout life (Burns, 2009, 2017; McFadden et al., 1996). However, hearing loss and/or ototoxic drugs may reduce or eliminate them (Probst, Lonsbury-Martin, & Martin, 1991), and there is some evidence of temporary changes induced by the menstrual cycle and use of oral contraceptives. Specifically, several studies suggested that OAEs fluctuate with the menstrual cycle, peaking around ovulation when levels of estradiol are high (Al-Mana, Ceranic, Djahanbakhch, & Luxon, 2010; Bell, 1992; Haggerty, Lusted, & Morton, 1993; Penner, 1995). However, in a recent study the evidence regarding OAE fluctuation during the menstrual cycle was not supported (McFadden, Pasanen, Maloney, Leshikar, & Pho, 2018). A temporary change in OAEs might be elicited by testosterone as well; a negative relationship between seasonal variations in testosterone and emission strengths was reported in male adult monkeys (McFadden, Pasanen, Raper, Lange, & Wallen, 2006). Thus, in addition to its prenatal effects on CEOAEs, testosterone also may exert dampening effects on emission amplitudes postnatally. Seasonal fluctuations in serum testosterone levels in adult men were found to correlate negatively with their CEOAE amplitudes as well (Snihur & Hampson, 2012b). In another study, Snihur and Hampson (2012a) suggested that estradiol also may be involved in regulating the production of OAEs, at least in the cochleae of females. They showed that women using oral contraceptives (that suppress endogenous fluctuations in estradiol) had “defeminized” weaker OAEs compared with a group of women undergoing a normal menstrual cycle (cf. McFadden, 2000). No association between differences in OAEs and circulating testosterone levels could be observed in the two female participant groups. Therefore, the authors concluded that their results might reflect differences in estradiol exposure with relatively higher levels of estradiol in normally cycling women resulting in more female-typical OAEs (Snihur & Hampson, 2012a).

Gender dysphoria (GD; American Psychiatric Association, 2013) is the significant distress or problems in functioning someone experiences due to an incongruence between the experienced/expressed gender and the assigned sex at birth. One hypothesis about the etiology of GD is that atypical levels of sex hormones during a critical period of sexual differentiation mediate a sex-atypical programming of certain localized regions of the brain and thereby the development of an atypical gender identity in transgender individuals (Bao & Swaab, 2011).

Due to the putative relationship between prenatal androgen exposure and CEOAE strength, CEOAE measurements have been used as a marker of prenatal androgen exposure. In a previous study, we indirectly investigated the relationship between the (atypical) development of gender identity and prenatal androgen exposure as estimated by CEOAE measurements (Burke, Menks, Cohen-Kettenis, Klink, & Bakker, 2014). Participants were 57 children and adolescents diagnosed with GD (24 had a male sex assigned at birth, self-identifying as female [referred to as “trans girls”], and 33 had a female sex assigned at birth, self-identifying as male [referred to as “trans boys”]). The terms “trans boys/girls” were chosen based on international guidelines saying “When employing references to a person’s assigned sex at birth, authors should use terms such as birth-assigned sex, or (if appropriate) legal sex, instead of natal male or natal female” (Bouman et al., 2017).

All participants were treatment-naïve and were either prepubertal or in early adolescence. The control participants were 65 boys (male sex assigned at birth) and 62 girls (female sex assigned at birth), all of whom had a gender identity congruent with their birth-assigned sex. In this prior study (Burke et al., 2014), it was indeed found that the group of 6–14-year-old (treatment-naïve) trans girls tended to have stronger, more female-typical CEOAEs compared to the male control group, while CEOAEs of trans boys were similar to those of the female controls. Also, in that study we replicated the previously observed sex differences in CEOAE response amplitudes with significantly stronger emissions in control girls than in control boys, but there were no sex differences between boys and girls with GD (Burke et al., 2014). These results, in line with several neuroimaging studies, suggest a less pronounced sexual differentiation in transgender individuals (Burke, Manzouri, & Savic, 2017, and for reviews, see Guillamon, Junque, & Gómez-Gil, 2016; Kreukels & Guillamon, 2016).

At the Center of Expertise on Gender Dysphoria at the VU University Medical Center, eligible adolescents diagnosed with GD may start using gonadotropin-releasing hormone analogs (GnRHa) in order to suppress pubertal maturation, and thus the irreversible development of the secondary sex characteristics of their sex assigned at birth (Kreukels & Cohen-Kettenis, 2011). From the age of 16 years on, as an important step in the gender-affirming treatment, adolescents with GD also may receive cross-sex hormones (CSH; testosterone for trans boys and estradiol for trans girls), in addition to their treatment with GnRHa, in order to develop secondary sex characteristics of their experienced gender (Delemarre-van de Waal & Cohen-Kettenis, 2006).

In the current study, based on the assumption that sex hormones also may exert activational effects (i.e., changes that can be both suppressing and enhancing) on CEOAEs postnatally, we investigated, using a cross-sectional design, whether hormonal interventions such as pubertal suppression and CSH treatment in transgender individuals affected their CEOAEs. We hypothesized that administration of estradiol in addition to suppression of endogenous testosterone production by means of GnRHa in trans girls would result in stronger emissions compared to controls matched on age and birth-assigned sex, similar to female-typical CEOAE response amplitudes. Conversely, administration of testosterone in addition to suppressing endogenous estradiol by means of GnRHa in trans boys was assumed to result in suppressed CEOAEs in trans boys compared to controls matched on age and birth-assigned sex. Lastly, we hypothesized that, for both groups, similar changes would occur with administration of GnRHa alone, although to a lesser extent. In a broader sense, this could provide additional information on the extent to which (suppression/addition of) sex hormones influence other physical characteristics of transgender people, in this case the inner ear, besides the previously observed effects on the secondary sex characteristics and the brain.

## Method

### Participants

A total of 81 boys and girls served as control participants, who were recruited via several primary and secondary schools in the Netherlands, and by inviting friends and relatives of the transgender participants. Out of these 81 controls, 66 had valid CEOAE recordings in both ears, nine were excluded from the left-ear analyses due to invalid left-ear recordings, and six participants were excluded from the right-ear analyses due to invalid right-ear recordings (see Statistical Analysis for the criteria of valid recordings). Thus, valid CEOAE data from 44 control boys and 37 control girls (Table 1) were used. These participants were divided into three groups age-matched to the corresponding GD groups: “early adolescent” age-matched to a group receiving no hormonal intervention (treatment-naïve), “mid-adolescent” age-matched to those receiving GnRHa, and “late-adolescent” age-matched to those receiving GnRHa plus CSH.

**Table 1:** Mean CEOAE amplitudes 1–4 kHz (in dB SPL) as a function of sex, condition, and ear

	Total sample^a^		Left-ear CEOAE^b^		Right-ear CEOAE	
	Age: *M* (SD), range	*N*	dB SPL: *M* (SD)	*N*	dB SPL: *M* (SD)	*N*
Trans girls^c^	15.9 (2.4), 11.0–20.0	43	10.7 (4.1)	37	12.1 (3.8)	40
Treatment-naïve	12.6 (0.9), 11.0–14.0	10		6		10
GnRHa	15.2 (1.0), 13.3–17.1	14		14		13
GnRHa + CSH	18.1 (0.8), 16.8–20.0	19		17		17
Trans boys^d^	15.6 (2.3), 10.3–20.3	62	12.3 (4.7)	59	12.5 (5.2)	60
Treatment-naïve	13.7 (2.4), 10.3–17.3	15		14		13
GnRHa^e^	15.0 (1.6), 12.3–18.0	26		25		26
GnRHa + CSH^f^	17.8 (1.1), 16.3–20.3	21		20		21
Control boys	14.5 (2.4), 10.8–18.3	44	12.4 (3.5)	37	12.2 (3.6)	40
Early adolescent	12.8 (1.9), 10.8–16.2	13		10		12
Mid-adolescent	13.9 (1.9), 10.8–15.8	18		15		16
Late adolescent	17.1 (0.8), 15.8–18.3	13		12		12
Control girls	14.8 (2.9), 10.8–18.5	37	14.1 (3.7)	35	15.5 (3.8)	35
Early adolescent	12.2 (1.7), 10.8–16.3	15		14		15
Mid-adolescent	15.1 (1.8), 11.5–16.4	10		9		10
Late adolescent	17.9 (0.4), 17.3–18.5	12		12		10

All control and treatment-naïve participants (except for three treatment-naïve trans girls) participated in our previous study (Burke et al., 2014)

^a^Total sample: All participants included in left-ear and/or right-ear analyses

^b^CEOAE = click-evoked otoacoustic emission

^c^Trans girls = individuals assigned male at birth

^d^Trans boys = individuals assigned female at birth

^e^GnRHa = gonadotropin-releasing hormone analog, puberty suppression

^f^CSH = cross-sex hormone treatment, estradiol for trans girls, testosterone for trans boys

A total of 106 children and adolescents diagnosed with GD were recruited at the Center of Expertise on Gender Dysphoria at the VU University Medical Center in Amsterdam. One participant was excluded due to invalid measurements in both ears. Out of the remaining 105 transgender participants, 91 had valid CEOAE recordings in both ears, nine were excluded from the left-ear analyses due to invalid left-ear recordings and five participants were excluded from the right-ear analyses due to invalid right-ear recordings. The 43 trans girls and 62 trans boys (Table 1) with valid left or right CEOAE data were divided into three groups according to their hormonal intervention: treatment-naïve, pubertal suppression by means of GnRHa administration or GnRHa plus CSH treatment. The latter is further referred to as the CSH group.

The treatment-naïve group consisted of 10 trans girls and 15 trans boys (*M*_age_ = 13.2, SD = 2.0, range 10.3–17.3; Table 1) who did not meet the criteria to start GnRHa treatment yet. The early adolescent control group consisted of 13 boys and 15 girls (*M*_age_ = 12.5, SD = 1.8, range 10.8–16.3).

The puberty-suppressed groups consisted of 14 trans girls and 26 trans boys (Table 1) who had been treated monthly with injections of 3.75 mg triptorelin (Decapeptyl-CR^®^, Ferring, Hoofddorp, the Netherlands) for, on average, 20.1 months (range 2–48 months), resulting in complete suppression of gonadal hormone production. These participants had not yet reached the age limit to start CSH treatment, but were expected to receive CSH in the future. The mid-adolescent control group consisted of 18 boys and 10 girls.

The CSH treatment groups consisted of 19 trans girls who received 17ß-estradiol (Progynova^®^, Bayer, Mijdrecht, the Netherlands or Cetura^®^, ACE Pharmaceuticals, Zeewolde, The Netherlands) on a daily basis for on average 22.7 months (range 5–47 months) and 21 trans boys who received a testosterone–ester mixture (Sustanon^®^ 250 mg/ml, Merck Sharp & Dohmebv, Oss, the Netherlands) every 2–4 weeks, for on average 11.8 months (range 2–28 months). CSH doses depended on the patient’s weight (trans girls) or body surface area (trans boys), and the starting dosage varied with the subject’s age. Until the age of 16.5 years, the starting dosage for estradiol was 5 µg/kg each day and for Sustanon^®^ 25 mg/m^2^ body surface area every 2 weeks. When older than 16.5 years, the dosage was 1 mg Progynova^®^/Cetura^®^ daily or 75 mg Sustanon^®^ every 2 weeks, respectively. All study participants who were treated with CSH also received a monthly triptorelin injection in order to suppress endogenous gonadal sex hormone production. They already had been receiving monthly triptorelin injections in order to suppress endogenous puberty before addition of CSH. The late-adolescent control group consisted of 13 boys and 12 girls.

In the present study, which aimed to focus on the hormone intervention effects on CEOAEs, we again included the data of the treatment-naïve transgender and control participants from our previous study (Burke et al., 2014). However, here, we selected only those transgender participants who were already in puberty, with a minimal tanner stage of 2 (Marshall & Tanner, 1969, 1970), in order to match the three hormonal-intervention groups with regard to their (previous) endogenous sex hormone exposure. Note that they all had some level of endogenous gonadal hormone exposure and were matched to controls on age and birth-assigned sex. For the current study, we included data only of those control participants who were above the age of 10, because all transgender participants were older than 10 years as well. Thus, in the current study pubertal (Tanner > 1) treatment-naïve transgender and cisgender control participants (age > 10), who also participated in our previous study (Burke et al., 2014), were included in addition to GnRHa- and GnRHa- plus CSH-treated participants with GD.

### Materials and Procedure

CEOAE recordings were performed with EZ-screen software and with an Otodynamics echo-port system ILO288, in combination with a laptop computer. The apparatus was calibrated each time it was put online for use. CEOAEs were recorded at five frequency bands (1000, 1414, 2000, 2828 and 4000 Hz) and in the Quick Screen (nonlinear) mode with a time window of 2.5–12.5 ms. CEOAE responses were measured in terms of dB SPL (decibel sound-pressure level). Each ear was tested for a fixed number of 250 clicks; the average emission response of the five frequency bands was used for further analyses. The click-stimulus input was set to approximately 80 (± 2.3) dB, which is in accordance with a clinical protocol for CEOAE recordings (Hall, 2000). A probe with an appropriately sized foam ear tip, thereby causing minimal discomfort for the participant, was placed in the external ear canal to seal the cavity completely. The probe fit was evaluated by the noise-level rejection meter: CEOAE data were regarded useful when environmental noise levels did not reach a threshold of 6 mPa. Participants were seated in a comfortable chair and were allowed to relax for a few moments prior to data collection. They were asked to relax their body and facial muscles during the recordings in order to ensure a low-noise measurement. Besides external noise, test order of the left and right ear also might influence the CEOAE recordings (Thornton, Marotta, & Kennedy, 2003); therefore, the right ear was tested first in each participant.

### Statistical Analysis

CEOAE recordings (the mean of the five frequency bands) with an amplitude of at least 0.99 dB SPL and a “whole-wave reproducibility” of more than 0.69 were used for analysis; whole-wave reproducibility was calculated as the correlation coefficient of interleaved nonlinear responses (Berninger, 2007). All recorded measurements were transferred to a database and analyzed using the Statistical Package for the Social Sciences, version 20 (SPSS Inc., Chicago, IL, USA).

Left- and right-ear emissions have previously been reported to differ in strength, with right-ear emissions being stronger than left-ear emissions, and sex differences were sometimes only observed in right-ear emission (Aidan, Lestang, Avan, & Bonfils, 1997; Driscoll, Kei, & McPherson, 2000; Ismail & Thornton, 2003; McFadden et al., 1996; Saitoh et al., 2006). Therefore, separate ANOVAs were conducted for each ear’s CEOAEs. An independent factorial analysis of variance (ANOVA) was used to analyze overall group differences in right CEOAE amplitudes, with right-ear CEOAE as dependent variable and condition (GD; control), sex assigned at birth (male; female) and age-group (1. treatment-naïve, early adolescent; 2. GnRHa, mid-adolescent; 3. GnRHa plus CSH, late adolescent) as independent variables. An identical independent factorial ANOVA approach was used to analyze differences in left CEOAE amplitudes.

Effects were considered statistically significant at *p* ≤ .05, and Bonferroni correction was applied post hoc to control for multiple comparisons. Cohen’s *d* was reported as an estimate of effect size for a mean difference between groups, where *d* was calculated as the difference between two means divided by the square root of the (weighted) mean of the variances corresponding to those two means (Cohen, 1988).

## Results

Demographic information for the subjects in all study groups is provided in Table 1. The Kolmogorov–Smirnov test and Levene’s test confirmed normality of the CEOAE data and that homogeneity of variance between groups could be assumed.

The transgender groups and their corresponding control groups did not differ in age, and the distribution of (trans) boys and (trans) girls was equal for all groups (Table 1). This was tested with planned contrasts using one-way ANOVA, which revealed that there was no significant difference between the mean age of the treatment-naïve group and the early adolescent control group, *t*(49.00) = 1.52, *p* = .134, the GnRHa group and the mid-adolescent control group, *t*(45.83) = 1.72, *p* = .093 and the CSH group and the late-adolescent control group, *t*(59.44) = 1.87, *p* = .066. Based on Pearson’s chi-square, there was no significant association between age-group and having a specific sex, *χ*^2^(5) = 6.42, *p* = .267.

### Gender Dysphoria versus Control Condition

**Fig. 1:**
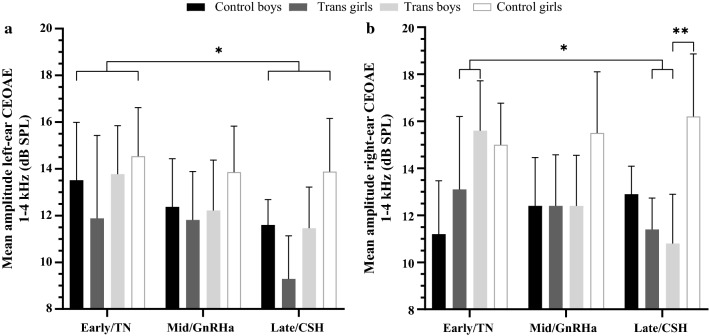
CEOAE response amplitude in the left (**a**) and right (**b**) ears of assigned-at-birth male and female control and GD groups for the three age/treatment groups. *Error bars* represent the 95% confidence interval. CEOAE, click-evoked otoacoustic emission; TN, treatment-naïve; GnRHa, gonadotropin-releasing hormone analog, puberty suppression; CSH, cross-sex hormone treatment; trans girls, female gender identity, male assigned at birth; trans boys, male gender identity, female assigned at birth; early/mid/late, early/mid/late-adolescent age. Pulled over sex and condition, the early/TN group had significantly stronger left-ear CEOAEs than the late/CSH group (*), right-ear CEOAEs were significantly weaker in participants with GD when receiving CSH (plus GnRHa) compared to treatment-naïve participants (*), and the CSH-receiving trans boys had significantly weaker right-ear CEOAEs than the late-adolescent control girls (**),*p ≤ .05; **p ≤ .01

Sex differences (across age-group) in right-ear CEOAE amplitudes were observed in the control condition, but not in the GD condition. In addition, whereas no differences between age-groups (i.e., treatment groups for GD) were found in controls, right-ear CEOAEs were significantly weaker in participants with GD when receiving CSH (plus GnRHa) compared to treatment-naïve participants (see Table 1 and Fig. 1b). This was tested with between-subject comparisons of a 2 (condition) × 2 (sex) × 3 (age-group) independent factorial ANOVA for right-ear CEOAEs, which revealed a significant main effect of sex, *F*(1, 163) = 9.00, *p* = .003, with overall stronger right-ear emissions in participants assigned female at birth than in participants assigned male at birth, as expected from the literature (McFadden et al., 1996). In addition, when pooling over age and condition there was a significant interaction between sex and condition, *F*(1, 163) = 4.09, *p* = .045; for control participants, females had stronger right-ear CEOAEs than males (normative sex difference), but trans boys and trans girls showed no differences in CEOAEs. Furthermore, a significant interaction was revealed between age-group and condition, *F*(2, 163) = 3.89, *p* = .022. Post hoc Bonferroni-corrected comparisons revealed that treatment-naïve transgender participants had significantly stronger right-ear CEOAEs compared to those receiving CSH (plus GnRHa) treatment, *p* = .016. There were no (age-group) differences between control participants. Right-ear emissions in control participants tended to be overall stronger than in transgender participants, but no significant main effect for condition was revealed, *F*(1, 163) = 3.42, *p* = .066. No significant interactions between sex and age-group, or sex, condition and age-group were observed.

Similarly, in left-ear CEOAEs there were differences between transgender participants and controls, and between males and females (see Table 1 and Fig. 1a). This was tested with a condition-by-sex-by-age-group independent factorial ANOVA for left-ear CEOAEs, which revealed a significant main effect of sex, *F*(1, 156) = 5.19, *p* = .024, with overall stronger left-ear emissions in participants assigned female at birth than in participants assigned male at birth, as expected (see Fig. 1a). There also was a significant main effect of condition, *F*(1, 156) = 5.27, *p* = .023, with overall stronger CEOAEs in the control condition than in the GD condition. A trend for a main effect of age-group was observed, *F*(2, 156) = 2.47, *p* = .088. Post hoc Bonferroni-corrected comparisons indicated overall, thus irrespective of condition, stronger left-ear CEOAEs in the treatment-naïve GD and early adolescent control participants compared to those receiving CSH treatment and late-adolescent controls, *p* = .014. Meaning the younger groups showed stronger CEOAEs than the older groups. No significant interactions between sex and condition, sex and age-group, condition and age-group or sex, condition and age-group were revealed.

### Hormone Intervention Effects

CSH-treated trans boys showed masculinized right-ear CEOAEs compared to late-adolescent control girls, in line with the hypothesized masculinizing effect of testosterone. However, no further statistically significant differences between groups with GD and matched control groups in either right-ear or left-ear CEOAEs were revealed (see Fig. 1). This was tested by a one-way ANOVA for right-ear CEOAEs in all participants assigned female at birth (trans boys, control girls), with age-group as an independent variable. There were significant differences between the three transgender groups and their age-matched control groups, *F*(5, 89) = 3.53, *p* = .006. Planned contrasts revealed no difference between treatment-naïve trans boys and early adolescent control girls, *d* = 0.15. Trans boys receiving GnRHa treatment tended to have weaker CEOAEs compared to mid-adolescent control girls, *t*(89) = − 1.75, *p* = .083, *d* = 0.61, and trans boys receiving GnRHa plus testosterone administrations (i.e., CSH group) had significantly weaker right-ear CEOAE amplitudes compared with the late-adolescent control girls, *t*(89) = − 3.00, *p* = .004, *d* = 1.16.

One-way ANOVA for right-ear CEOAEs in all participants assigned male at birth (trans girls, control boys), using age-group as an independent variable, did not reveal any significant differences between the three groups with GD and their age-matched control groups. However, in the right ear, treatment-naïve trans girls tended to have stronger, thus sex-atypical CEOAEs than the early adolescent control boys, *d* = 0.41, while trans girls receiving GnRHa had similar CEOAEs as the mid-adolescent control boys, thus sex-typical, *d* = 0.01. Notably, a moderate effect size indicated that trans girls receiving GnRHa plus estradiol administrations had weaker, thus even exaggerated male-typical CEOAE amplitudes, when compared with late-adolescent control boys, *d* = 0.62.

Two one-way ANOVAs for left-ear CEOAEs among those participants with the same birth-assigned sex, investigating group differences between (1) trans boys and control girls, and (2) trans girls and control boys, yielded no significant group differences for either sex (see Fig. 1a). However, in the left ear, moderate-to-large effect sizes indicated that trans boys receiving GnRHa plus testosterone administrations had weaker CEOAEs than the late-adolescent control girls, *d* = 0.60, and that trans girls receiving GnRHa plus estradiol administrations, again had weaker, exaggerated male-typical CEOAEs than the late-adolescent control boys, *d* = 0.76 (see Fig. 1a). In addition, small effect sizes indicated weaker left-ear CEOAEs in treatment-naïve trans girls than in early adolescent control boys, *d* = 0.39, and, as expected, weaker CEOAEs in trans boys receiving GnRHa therapy compared to mid-adolescent control girls, *d* = 0.37.

## Discussion

In the present study, we examined whether CEOAE response amplitudes in adolescents diagnosed with GD differed as a function of their hormone treatment when compared to (birth-assigned) sex- and age-matched controls, and thus whether CEOAEs were affected postnatally by circulating sex hormones. In accordance with past demonstrations of the suppressive effects of androgens on CEOAEs (McFadden, 2008; McFadden et al., 2009), trans boys (female sex assigned at birth) who received testosterone treatment showed significantly weaker right-ear CEOAEs compared with age-matched control girls. Thus, testosterone administration seemed to dampen or “defeminize” CEOAE response amplitudes. Note that participants receiving testosterone (or estradiol) always received GnRHa as well, beginning before the start of CSH treatment. Therefore, this effect may be attributed to the addition of exogenous hormones as well as to the (long-term) suppression of endogenous hormones. As can be inferred from Fig. 1, pubertal suppression with GnRHa in trans boys tended to masculinize their CEOAEs, whereas a reverse effect of more feminized/demasculinized CEOAEs in the trans girls was not apparent. Moreover, contrary to our expectations that estradiol would exert enhancing effects on emission amplitudes, right-ear CEOAEs in trans girls receiving estradiol administrations (in addition to GnRHa) were not different from those of the age-matched control boys. Thus, testosterone as well as estradiol (plus GnRHa) treatment had masculinizing, activational effects on right-ear CEOAEs, as suggested previously by Snihur and Hampson (2012a, 2012b).

Left-ear CEOAEs were not different for three treatment groups with GD and their age- and sex-matched control groups. This is in line with previous findings that left- and right-ear emissions differ in strength, with right-ear emissions being stronger than left-ear emissions and that sex differences were sometimes only observed in right-ear emissions (Aidan et al., 1997; Driscoll et al., 2000; Ismail & Thornton, 2003; McFadden et al., 1996; Saitoh et al., 2006). As in our previous study (Burke et al., 2014), we will therefore further focus on the right-ear CEOAE results.

We confirmed a significant main effect of sex, indicating overall stronger right-ear CEOAEs in (at-birth-assigned) females than in males. However, a significant interaction effect of sex and condition also indicated that this typical sex difference pattern was not similarly observed for control and GD participants alike. Hence, across treatment groups, no differences in CEOAEs between trans girls and trans boys were found. More specifically, whereas treatment-naïve trans boys had sex-typical right-ear CEOAEs (compared with control girls; *d* = 0.15), those of the treatment-naïve trans girls tended to be increased relative to age-matched control boys, thus atypical for their sex assigned at birth (*d* = 0.41). This finding was not unexpected, since the treatment-naïve groups of the current study (*N* = 10 trans girls; 11–14 years of age) were a sub-sample selected from a larger group of pre- and early adolescent participants (*N* = 24 trans girls; 6–14 years of age) of our prior study (Burke et al., 2014). In that study also, the larger group of 24 trans girls showed relatively, though not significantly, feminized right-ear CEOAEs compared with an age-matched cis-male control group (*d* = 0.48). Although speculative, but partially in support of a prominent hypothesis on the etiology of GD (Bao & Swaab, 2011; Swaab, Chung, Kruijver, Hofman, & Ishunina, 2002), our findings in the youngest, treatment-naïve groups suggest a hypomasculinized early sexual differentiation in trans girls and a typically female early sexual differentiation in trans boys. However, due to the small sample sizes in our study, effects could as well be due to larger variability and should therefore be confirmed in larger samples.

### Effects of Testosterone on Click-Evoked Otoacoustic Emissions

In trans boys, testosterone treatment in addition to GnRHa had suppressive effects on their CEOAE response amplitudes when compared to late-adolescent control girls. To the best of our knowledge, this is the first study that investigated the effects of postnatal androgen administration on CEOAEs in trans boys. Postnatal androgen effects on CEOAEs in males previously have been suggested by studies in rhesus monkeys (McFadden et al., 2006) and men (Snihur & Hampson, 2012b). Associated with seasonal androgen fluctuations, CEOAEs in rhesus monkeys appeared to be weaker in winter time, when circulating testosterone levels were high (McFadden et al., 2006). Similarly, CEOAEs in men correlated negatively with monthly fluctuations in blood testosterone levels (Snihur & Hampson, 2012b). Of note, trans boys receiving testosterone administrations already had been receiving GnRHa, which means that differences might not be solely attributed to (the addition of) testosterone. Ideally, future studies should include an additional group of late-adolescent trans boys, receiving GnRHa only, to address this issue. Our findings thus provide evidence for the dampening influences of a combination of pubertal suppression and postnatal androgens on CEOAEs.

### Effects of Estradiol on Click-Evoked Otoacoustic Emissions

We found that estradiol administrations in addition to GnRHa had no significant effects on right-ear CEOAEs in trans girls when compared to late-adolescent control boys. In contrast, several previous studies had suggested that relatively high levels of estradiol, such as during ovulation, correlated positively with OAE frequency and amplitude during the menstrual cycle (Al-Mana et al., 2010; Haggerty et al., 1993; Penner, 1995). In addition, McFadden, Pasanen and Callaway (1998) found a higher frequency of SOAEs in a trans woman after treatment with estrogen as compared to before the treatment. However, our findings did not support the hypothesis of enhancing effects of estradiol on CEOAEs. Moreover, the moderate effect size (*d* = 0.62) comparing trans girls receiving GnRHa plus estradiol and late-adolescent control boys indicated even weaker, rather than the expected higher amplitudes in the trans girls. This result, however, may be in line with two previous studies by McFadden (2000) and Snihur and Hampson (2012a) who showed that women using oral contraceptives had significantly weaker OAEs compared with normally cycling women. Likewise, the trans girls in our study receiving daily estradiol administrations as part of their CSH treatment showed suppressed CEOAEs compared to age-matched control boys. These “masculinizing” effects of oral contraceptives on the auditory system have previously been suggested by Elkind-Hirsch, Stoner, Stach and Jerger (1992) and McFadden (2000), who found that OAEs and several auditory brainstem response measures were “defeminized” in women using oral contraceptives in comparison with female non-users. Also, as has previously been suggested by Haggerty et al. (1993) and Penner (1995), female-typical fluctuations in OAE amplitude and frequency seem to be dependent on a cyclical, pulsatile pattern of estradiol secretion. Accordingly, our results could be explained by the notion that a continuous administration of estradiol (as in both oral contraceptive use and CSH administration) results in more male-typical, thus weaker OAEs.

However, in a recent study by van Hemmen, Cohen-Kettenis, Steensma, Veltman and Bakker (2017) no differences in CEOAEs between women using oral contraceptives and normally cycling women were found. An alternative explanation for the male-typical CEOAEs in trans girls may be that, in an (at-birth-assigned) male organism/body part (e.g., the inner ear), estradiol and testosterone show very similar activational effects. In support of this, several animal studies (reviewed in Baum, 2003; Bakker et al., 2006; Jost, 1983) have suggested that both testosterone and estradiol may masculinize or defeminize the nervous system and elicit male-typical sexual behavior in adult, previously castrated, rodents. There is little evidence for a role of fetal estradiol in male-typical psychosexual differentiation, because men who have either a point mutation in the estradiol receptor gene or lack the aromatase enzyme have a male gender identity (reviewed in Baum, 2006). Nevertheless, there is evidence for activational effects of estradiol on the brain in adult men. Indeed, a large clinical study (Finkelstein et al., 2013) showed that treatment with an aromatase inhibitor led to a significant decline in sexual desire in adult men. Feminizing effects of estradiol have been suggested by a study that found that monozygotic female twins exhibited more SOAEs than dizygotic or non-twin females (McFadden & Loehlin, 1995). Although speculative, this might be indicative of a positive association between prenatal estradiol exposure and feminized SOAEs. But again, it should be noted that in the current study differences may not merely be attributable to estradiol, because late-adolescent trans girls also were receiving GnRHa before and during estradiol treatment.

Taking these findings together, both testosterone and estradiol (in addition to GnRHa) seem to be actively implicated in facilitating or inhibiting the cochlear amplification mechanism and may thus actively “feminize” or “masculinize” OAEs. It should be noted that the influence of GnRHa only on CEOAEs in late-adolescent participants with GD is unknown. Therefore, the effects in the CSH groups might be attributable to addition of estradiol/testosterone, a long-term GnRHa treatment, or an interaction between the two. Also, of note, both the possible testosterone-mediated dampening effects on CEOAEs in our trans boys and the weaker emissions in trans girls receiving GnRHa plus estradiol administration may be explained by an estradiol-driven effect. Dependent on the target tissue, testosterone either may have a direct effect by binding to the androgen receptor or may be locally converted to estradiol by the enzyme aromatase and consequently bind to ERs to exert its effect.

Furthermore, testosterone and estradiol may exert their effects on OAEs during different time windows. The outer hair cells in the rodent as well as the human cochlea have been shown to contain the receptor types ERα and ERβ (Hultcrantz, Simonoska, & Stenberg, 2006; Motohashi et al., 2010; Stenberg et al., 2001; Stenberg, Wang, Sahlin, & Hulcrantz, 1999). In rats, both receptor types were reported to be up- or down-regulated dependent on different postnatal developmental stages, whereas no ER expression was observed during fetal development (Simonoska, Stenberg, Masironi, Sahlin, & Hultcrantz, 2009), suggesting that any estrogen-sensitive mechanisms associated with auditory functioning may occur during postnatal life.

Thus, the dampening effects of the testosterone (plus GnRHa) treatment on CEOAEs in our trans boys may reflect a physiological estradiol-mediated mechanism, whereas the weaker emissions following estradiol treatment in the trans girls may be explained by similar activational effects of estradiol and testosterone on the male ear. Furthermore, testosterone seems to suppress CEOAEs both endogenously and exogenously, whereas estradiol seems to only enhance CEOAEs endogenously.

### GnRH Action and Quiescence

Trans boys receiving GnRHa (which suppressed any endogenous gonadal hormone production) had somewhat weaker, though not statistically significant, CEOAE response amplitudes compared with the age-matched control girls. This is in line with the assumed enhancing effects of endogenous estradiol on CEOAEs in (birth-assigned) females (e.g., during the menstrual cycle) (Bell, 1992; Haggerty et al., 1993; Penner, 1995).

In contrast, GnRHa administration in trans girls, thus suppressing the effects of endogenous testosterone, did not elevate their CEOAEs. However, we previously showed, and confirmed in the current study, that treatment-naïve trans girls already exhibit slightly stronger, more female-typical emission amplitudes, compared with control boys (Burke et al., 2014). Therefore, potential CEOAE enhancing effects of testosterone suppression might be relatively smaller in trans girls, because emission amplitudes in treatment-naïve trans girls were increased a priori (see Fig. 1b).

During childhood, the hypothalamic-pituitary–gonadal (HPG) axis is virtually quiescent until it is reactivated at the onset of puberty (Grumbach, 2002; Nathan & Palmert, 2005). In male fetal development, by the end of the first trimester of pregnancy (Grumbach, 2002), the hypothalamus starts to produce GnRH, which stimulates the production of gonadotropins and gonadal sex steroids. During the first few weeks after birth, GnRH secretion again increases significantly in males, resulting in a second, postnatal testosterone surge between 1 and 3 months of life, followed by a gradual decrease to prepubertal levels by 4–6 months (Finegan, Bartleman, & Wong, 1989; Waldhauser, Weissenbacher, Frisch, & Pollak, 1981). In females, the ovaries are relatively quiescent prenatally, but female infants show a similar, though somewhat later postnatal activation of the HPG axis as boys. High levels of estradiol are secreted by the ovaries during 6–12 months after birth, which start to decline by 12 months of age, but continue until the age of 24 months (Grumbach, 2002; Quigley, 2002; Waldhauser et al., 1981). Interestingly, relatively weaker CEOAEs in girls and less distinct sex differences in emission strengths have been observed in pediatric (2–6 years of age) populations (Kapoor & Panda, 2006; Lamprecht-Dinnesen et al., 1998) during childhood quiescence of the HPG axis. Neonates and infants during the first year of age, in contrast, have been reported to show significant sex differences in OAEs, similar to adult populations (Collet, Gartner, Veuillet, Moulin, & Morgon, 1993; Kapoor & Panda, 2006; Lamprecht-Dinnesen et al., 1998; Strickland et al., 1985). Therefore, sex differences in OAE frequency and amplitude are most distinct during periods of GnRH secretion, and thus during gonadal hormone action. Vice versa, in the current study, we showed that suppressing gonadal hormones by means of GnRHa indeed resulted in weaker CEOAEs.

Our results should be viewed in light of some limitations that may be addressed by future studies. Because we conducted cross-sectional comparisons, no inferences regarding developmental changes in CEOAEs associated with the hormonal interventions in individuals with GD could be made. Therefore, prospective studies following transgender adolescents during the different phases of hormonal intervention (prior to any intervention, during pubertal suppression and CSH treatment) should provide more direct evidence for the hypothesized relationship between gonadal hormone action and CEOAE amplitudes. Furthermore, the groups with GD should be compared to control groups without GD, not only matched with regard to age but also with regard to pubertal status, and to an additional late-adolescent GD group receiving GnRHa only.

In conclusion, our findings show that CEOAEs may not be used as an unequivocal measure of prenatal androgen exposure as they can be modulated postnatally by sex hormone exposure, in particular in puberty, but also during hormonal treatment. We propose that postnatal variations in CEOAE amplitude may be mediated by estradiol-regulated mechanisms.
